# Generation of a recombinant Saffold Virus expressing UnaG as a marker for the visualization of viral infection

**DOI:** 10.1186/s12985-023-02142-8

**Published:** 2023-08-07

**Authors:** Takako Okuwa, Toshiki Himeda, Koichi Utani, Masaya Higuchi

**Affiliations:** Department of Microbiology, Kanazawa Medical University School of Medicine, 1-1 Daigaku, Uchinada, Ishikawa 920-0293 Japan

**Keywords:** Saffold virus, UnaG

## Abstract

**Background:**

Saffold virus (SAFV), which belongs to the genus *Cardiovirus* of the family *Picornaviridae*, is associated with acute respiratory or gastrointestinal illnesses in children; it is also suspected to cause severe diseases, such as acute flaccid paralysis and aseptic meningitis. However, the understanding of the mechanism of its pathogenicity is still limited due to the many unknowns about its lifecycle; for example, the cellular receptor for its infection remains to be determined. A system to monitor SAFV infection in vitro and in vivo is required in order to accelerate research on SAFV.

**Results:**

We generated a recombinant SAFV expressing green fluorescent protein (GFP) or UnaG, a novel fluorescent protein derived from Japanese eel. HeLa cells infected by either GFP or UnaG-expressing SAFV showed a bright green fluorescent signal, enabling convenient monitoring of SAFV infection. However, the expression of GFP but not UnaG was quickly lost during virus passaging due to the difference in genetic stability in the SAFV virus genome; the UnaG gene was stably maintained in the virus genome after at least five passages.

**Conclusions:**

SAFV infection of cultured cells can easily be monitored using UnaG-expressing SAFV, which is superior to GFP in terms of genetic stability in the virus genome. This virus could be a useful tool for SAFV research, such as comparing the susceptibility of various cells to SAFV infection and evaluating the effects of antivirals on SAFV infection in high-throughput screening.

**Supplementary Information:**

The online version contains supplementary material available at 10.1186/s12985-023-02142-8.

## Background

Saffold virus (SAFV) belongs to the genus *Cardiovirus* of the family *Picornaviridae*, which is a small, non-enveloped, and icosahedral particle with a positive-sense single-stranded RNA genome. SAFV was discovered as the first human cardiovirus in 2007 in the stool of an infant presenting with fever of unknown origin in 1981 [1]. Since then, the detection of SAFV in children suffering from either acute respiratory or gastrointestinal illnesses has been reported [2–6]. Additionally, SAFVs have been detected in samples from patients with severe illness (e.g., acute flaccid paralysis, aseptic meningitis, myocarditis, acute pancreatitis and cerebellitis) [7–11]. However, the pathogenicity of SAFV, which causes a variety of mild to severe symptoms, remains unclear. The use of a recombinant SAFV harboring a reporter gene, such as green fluorescent protein (GFP), which enables convenient and real-time monitoring of SAFV replication in vitro and in vivo, would be very beneficial for elucidating the mechanism of SAFV pathogenicity.

The SAFV genome consists of a long open reading frame (ORF), 5’ and 3’ untranslated regions (UTR), and a variable length of poly (A) tail at the 3’ UTR. The ORF encodes a leader protein (L), four capsid proteins (VP1 to VP4) and seven non-structural proteins (2 A, 2B, 2 C, 3 A, 3B, 3 C, and 3D) [1]. Polyprotein is translated from the ORF, and then processed by the 3 C protease into mature viral proteins post-translationally, whereas the co-translational separation of the polyprotein occurs at the StopGo motif. This motif is oligopeptide D(V/I) ExNPG|P (where the “|” represents the junction between 2 A and 2B) mediating the StopGo process [12, 13], and is conserved in the 2 A/2B junction of SAFV (DIETNPG|P) [14]. The StopGo process has also been referred to as “ribosomal skipping” or “Stop-Carry On” and prevents the formation of a peptide bond between glycine and proline, but allows for the continuation of translation.

To advance the molecular pathogenetic studies of SAFV using reverse genetics, we previously established an infectious cDNA clone of SAFV-3 (the JPN08-404 strain) [8]. In the present study, based on this cDNA clone, we generated two recombinant SAFVs expressing a reporter gene, GFP or UnaG, to readily detect infection in living cells by inserting them between 2 A and 2B using the StopGo motif. UnaG is a novel green fluorescent protein derived from Japanese eel [15], which, at 139 amino acids, is smaller in size in comparison to GFP (239 amino acids). In the present study, we demonstrate that UnaG is a superior marker for SAFV infection to GFP with respect to the genomic stability of the virus genome. We further show the utility of UnaG-expressing SAFV for the study of SAFV infection and the screening of anti-viral drugs.

## Results and discussion

### Establishment of a recombinant SAFV-3 expressing a reporter gene

**Fig. 1:**
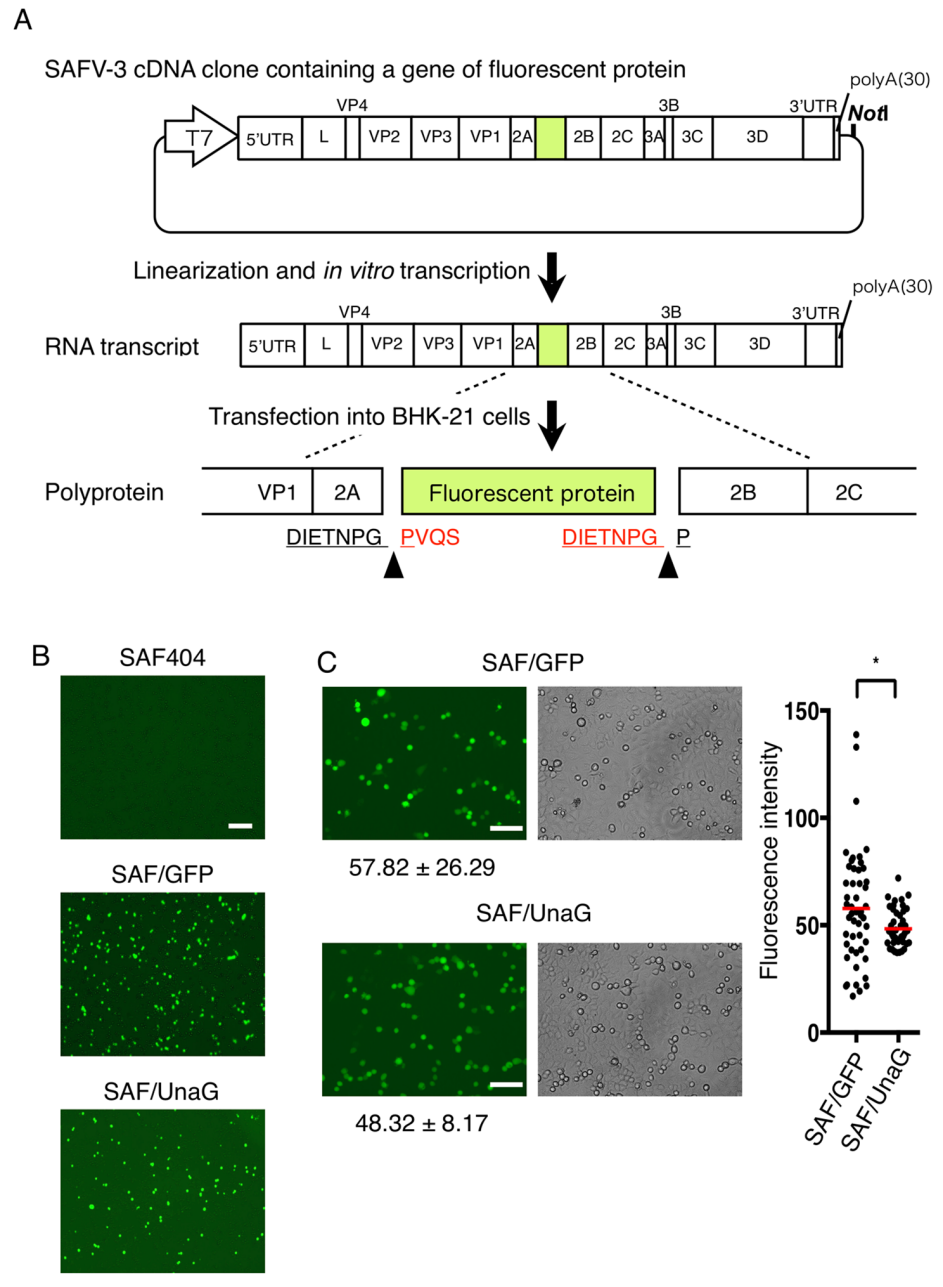
Generation of reporter-expressing SAFV-3. (**A**) A schematic diagram of the insertion of fluorescent protein into the SAFV genome and the generation of the reporter-expressing virus. A fluorescent protein gene (UnaG or GFP) with StopGo motifs at the 5’ and 3’ ends was inserted between 2 A and 2B of pSAF404. The resulting constructs (pSAF/UnaG and pSAF/GFP) were linearized with *Not*I and RNA was synthesized from the T7 promoter by in vitro transcription. Arrowheads indicate the separation site by StopGo during translation. Underlined peptides represent StopGo motifs. Peptide sequences added to both ends of the fluorescent protein are shown in red. (**B**) BHK-21 cells transfected with RNA transcripts synthesized from pSAF404, pSAF/GFP, and pSAF/UnaG by CUGA 7 RNA polymerase. At 20 h post-transfection, cells and supernatants containing recombinant viruses were harvested. Images of transfected cells were photographed using a JULI™ smart fluorescent cell analyzer before harvesting. Scale bar, 200 μm. (**C**) HeLa cells infected with P0 SAF/GFP or SAF/UnaG viruses were photographed at 16 h post-infection. The values of mean fluorescence intensity and SD are represented. Scale bar, 100 μm. The graph represents the fluorescence intensity of 50 cells. The red horizontal bars indicate the means. Statistical analysis was performed using the nonparametric Mann Whitney test (*p = 0.0418)

To generate a reporter-expressing recombinant SAFV genotype 3 (SAFV-3), we inserted a GFP or UnaG gene, with two StopGo motifs at both 5’ and 3’ ends, into the full-length cDNA of SAFV-3 (JPN08-404 strain) [8]. This should enable the translation of fluorescent proteins without any loss of viral proteins. We considered the insertion of reporter genes between 2A and 2B using the StopGo motif to be superior to other methods, such as inserting them into 3’ UTR or 5’ UTR, for the following reasons: Firstly, the precise sequence recognized by the SAFV 3 C protease, necessary for separating reporter proteins from the viral polyprotein when inserted into the 3’ UTR or 5’ UTR, was unknown. Secondly, inserting the reporter genes into 3’ UTR could potentially lead to reduced production of reporter proteins, as translation of viral proteins downstream of 2B is low in cardioviruses [16, 17]. We designated these cDNA clone of GFP- and UnaG-expressing recombinant SAFV-3 as pSAF/GFP and pSAF/UnaG, respectively (Fig. 1A). Then, the infectious RNA was i*n vitro* transcribed from *Not*I-linearized pSAF/GFP and pSAF/UnaG, and transfected into BHK-21 cells to generate the GFP- and UnaG-expressing recombinant SAFV-3 (SAF/GFP and SAF/UnaG). The transfected cells clearly showed a comparable expression of GFP and UnaG and a comparable cytopathic effect (CPE) (Fig. 1B and data not shown). To confirm the production of infectious recombinant viruses, we inoculated HeLa cells with cell culture supernatants from infectious RNA-transfected BHK-21 cells that contained passage 0 (P0) viruses. The inoculated HeLa cells showed the expression of GFP and UnaG, along with the appearance of CPE, thereby confirming the production of infectious SAF/GFP and SAF/UnaG (Fig. 1C). When comparing the fluorescence intensity of infected cells, SAF/UnaG-infected cells displayed a slightly lower mean fluorescence intensity value (48.32) compared to SAF/GFP-infected cells (57.82). However, they showed more consistent signals than GFP, which is considered advantageous when determining infection using fluorescence microscopy. Thus, these results demonstrated that UnaG as well as GFP can be used as a fluorescent reporter protein to visualize SAFV-3 infection.

### Stability of inserted reporter genes in SAFV-3 genome

**Fig. 2:**
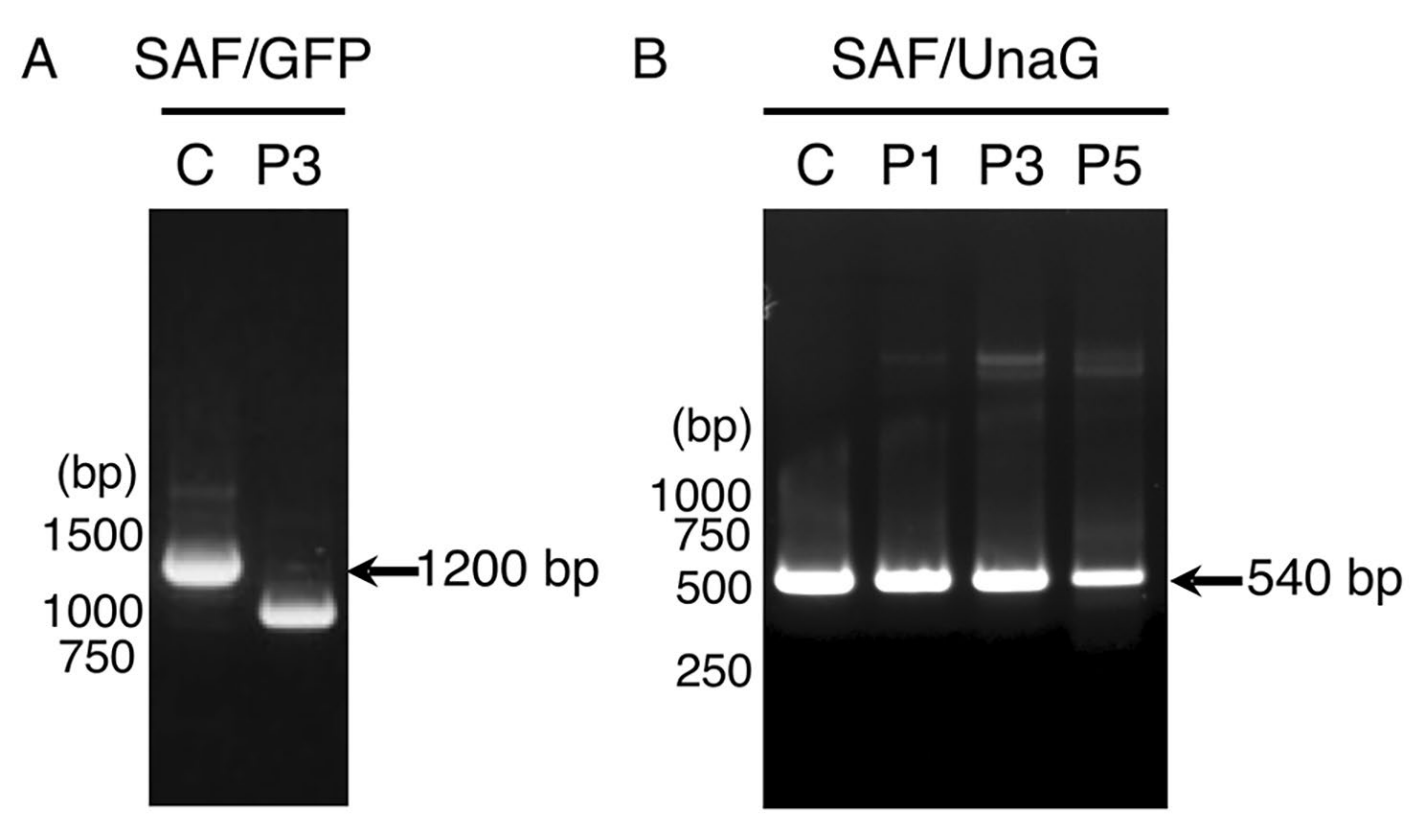
Genetic stability of SAF/GFP and SAF/UnaG genomes. To examine the genetic stability of GFP (**A**) and UnaG (**B**) genes in the SAFV-3 genome, SAF/GFP or SAF/UnaG viruses were serially passaged in Hela cells. Genomic RNAs were extracted from the clarified supernatants of SAF/GFP (P3) and SAF/UnaG (P1, P3 and P5), followed by RT-PCR. The full-length inserts were amplified using pSAF/GFP and pSAF/UnaG as templates (lane **C**). The positions of the size markers are indicated to the left of each panel

During the passaging process for the propagation of SAF/GFP and SAF/UnaG virus stocks, we observed a quick loss of the GFP expression but not the UnaG expression in infected cells (data not shown). We attributed the loss of the GFP signal during passaging to the loss of the GFP gene in the genome. Thus, we investigated the genetic stability of GFP and UnaG genes in the SAFV-3 genome by RT-PCR. Following the initial recovery of the P0 SAF/GFP and SAF/UnaG viruses, we serially passaged these viruses five times in HeLa cells and prepared P1, P3, and P5 SAF/UnaG, and P3 SAF/GFP virus stocks. RT-PCR of these stocks revealed that the exogenous GFP gene was unstable and shed from the SAFV-3 genome; the full length GFP was not detected at all after three passages (Fig. 2A). In contrast, the UnaG gene was stable and retained within the SAFV-3 genome through at least five passages (Fig. 2B). These results indicated that recombined GFP-null SAFV-3 emerged and outcompeted SAF/GFP during passaging, probably due to the growth disadvantage of SAF/GFP. On the other hand, the relatively smaller size of UnaG might contribute to its being stably retained in the SAFV-3 genome during passaging. This is consistent with a previous report showing that mengovirus, another cardiovirus, can stably harbor exogenous genes of < 500–600 bases, when inserted between 2A and 2B with similar StopGo motifs [18].

### Growth kinetics of SAF/UnaG in HeLa cells

**Fig. 3:**
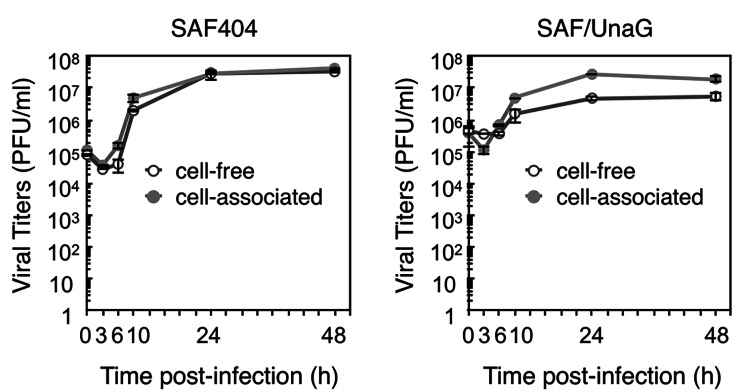
Growth curve of SAF/UnaG in HeLa cells. HeLa cells were infected with wild type recombinant SAFV-3 (SAF404) or SAF/UnaG at an MOI of 5. The culture supernatants (open circle) and cells (closed circle) were collected at the indicated times and titrated by a standard plaque assay on HeLa cells. Titers shown are the mean ± standard deviation in three independent experiments

To investigate whether the insertion of the exogenous UnaG gene into the virus genome would affect the efficiency of virus replication, the growth kinetics of SAF/UnaG virus was analyzed by a standard plaque assay using HeLa cells (Fig. 3). The titer of SAF/UnaG virus reached its peak at 24 h post-infection, similar to the wild-type recombinant SAFV-3 (SAF404) virus. Moreover, the growth kinetics of SAF/UnaG virus resembled previous data obtained from the parental strain JPN08-404 and cDNA-derived SAF404 [8]. Thus, these results demonstrated that the insertion of a reporter gene between 2 A and 2B with StopGo motifs does not perturb the replication and infection cycle of the recombinant SAFV-3. Taken together with the stability of UnaG gene in SAFV-3 genome, these results indicated that the UnaG-expressing recombinant SAFV-3 could be a useful tool for the visualization of SAFV-3 infection in experimental settings.

### SAFV-3 susceptibility of cell lines determined by SAF/UnaG

**Fig. 4:**
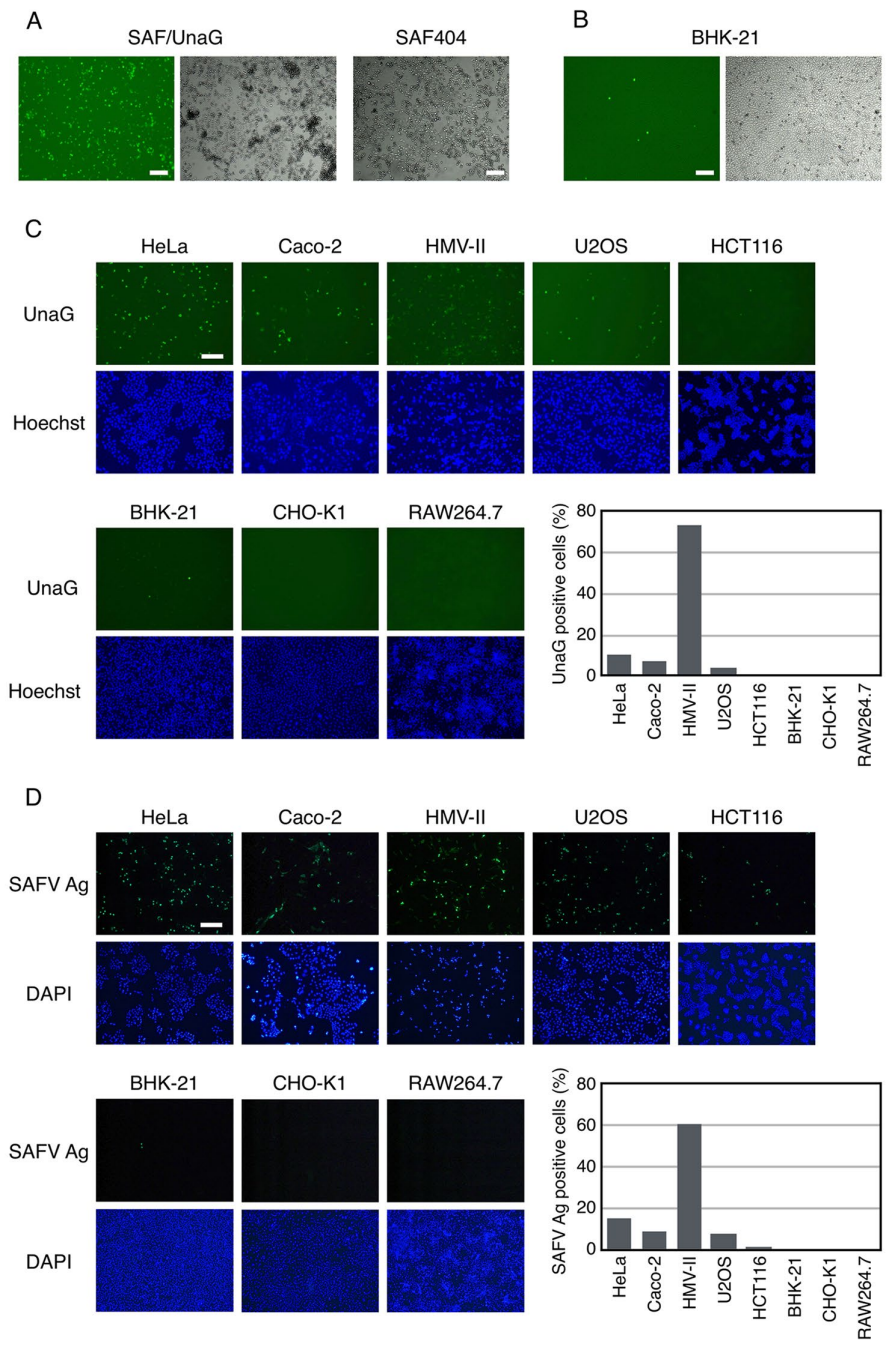
Susceptibility of cell lines to SAFV infection. (**A**) HeLa cells were infected with SAF/UnaG or wild-type recombinant SAFV-3 (SAF404) at an MOI of 10. UnaG fluorescence (green, left panel) and the CPE were photographed at 24 h post-infection. (**B**) BHK-21 cells were infected with SAF/UnaG at an MOI of 10. UnaG fluorescence (green, left panel) and CPE were photographed at 24 h post-infection. (**C** and **D**) Cells were infected with SAF/UnaG (C) or SAF404 (D) at an MOI of 1 and were photographed (C) or stained with anti-SAFV3 antiserum (D) at 16 h post-infection for HeLa, Caco-2, and HMV-II cells, and at 50 h post-infection for U2OS, HCT116, BHK-21, CHO-K1, and RAW264.7 cells. The percentage of infected cells was determined after examining at least 800 cells. Scale bar, 200 μm

To verify the utility of SAF/UnaG, we first infected HeLa and BHK-21 cells with SAF/UnaG and compared the CPE and fluorescent signals in these cells. Our previous studies have reported that HeLa cells are highly susceptible to SAFV-3 infection [19, 20]. When HeLa cells were infected with SAF/UnaG virus, the CPE was observed to be similar to that of the wild-type virus and UnaG fluorescence signal in infected cells (Fig. 4A). Although BHK-21 cells transfected with the infectious RNA of SAFV-3 were able to produce viral progeny (Fig. 1B), BHK-21 cells inoculated with the SAF/UnaG virus showed only sporadic fluorescent cells and no increase in the expression of UnaG or the CPE, indicating that SAFV-3 did not infect BHK-21 cells (Fig. 4B). Thus, BHK-21 cells are unsusceptible to SAFV-3 infection, probably due to the absence of SAFV receptors.

We further investigated the susceptibility of other human and rodent cell lines by infecting them with an equal amount of SAF/UnaG. or SAF404 and evaluating the expression of UnaG or SAFV antigen (Ag), respectively. The percentage of UnaG-positive cells showed a strong correlation with that of SAFV Ag-positive cells (Fig. 4 C **and D**), indicating that the SAF/UnaG virus can serve as a viable substitute for SAFV in evaluating the efficiency of SAFV infection. Among the human cell lines tested, Caco-2, U2OS, and HMV-II exhibited susceptibility, with HMV-II cells displaying the highest susceptibility. Conversely, we observed minimal UnaG expression but detected a small number of SAFV Ag-positive cells in HCT116 cells (Fig. 4 C **and D**), suggesting their low susceptibility to SAFV-3. The discrepancy between UnaG and SAFV Ag may be attributed to the higher sensitivity of SAFV Ag detection through immunofluorescence in cells with low susceptibility. In rodent cell lines RAW264.7 and CHO-K1, UnaG-expressing cells were sporadically observed, but the expression of the UnaG and the SAFV Ag did not spread to the surrounding cells (Fig. 4 C **and D**). These results indicated that rodent cells are not susceptible to SAFV-3 infection and that there is a difference in SAFV-3 susceptibility among human cell lines. It would be interesting to know whether this difference in human cells is due to differences in the expression of viral infection receptors, which are currently unidentified, or host factors involved in other processes such as viral replication.

### Evaluation of the antiviral effect of pleconaril on SAFV-3 infection using SAF/UnaG

**Fig. 5:**
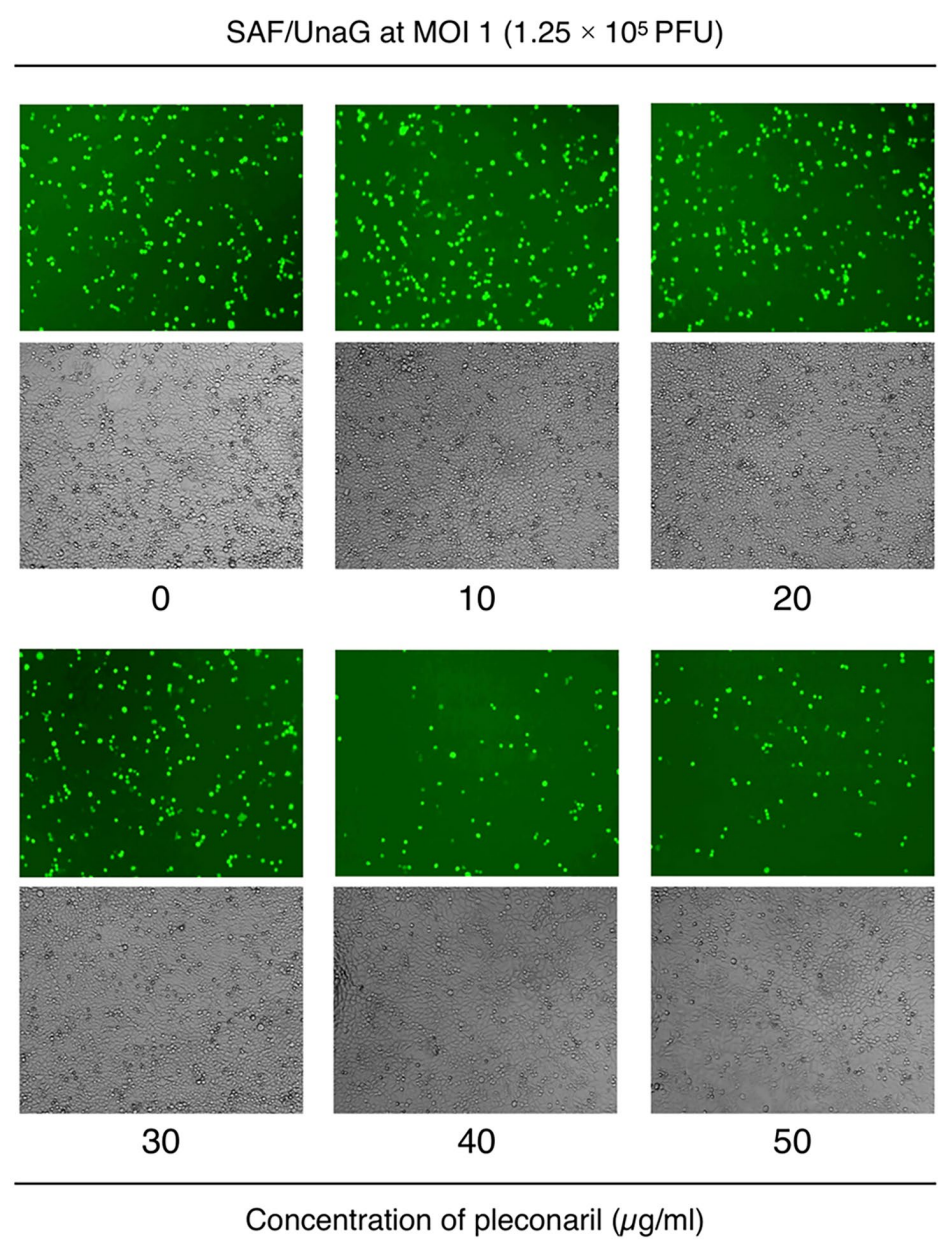
Antiviral effect of pleconaril on SFV/UnaG infection. SAF/UnaG were pretreated with indicated concentrations of pleconaril for 1 h at room temperature. HeLa cells were infected with the pretreated SAF/UnaG at an MOI of 1 (1.25 × 105 PFU/well). Cells were photographed at 19 h post-infection. The antiviral effect of pleconaril was evaluated by the number of UnaG-expressing cells at 19 h post-infection. No cytotoxicity of DMSO or pleconaril was observed in any of the wells

We next examined the potential use of SAF/UnaG for antiviral drug discovery research using pleconaril as a model. Pleconaril is an anti-picornaviral agent that binds to the hydrophobic pocket located in the capsid protein VP1, thereby preventing viral attachment to host cells and conformational changes necessary for viral uncoating in enteroviruses and rhinoviruses [21]. Pleconaril shows broad-spectrum activity against enteroviruses and rhinoviruses, but the sensitivity of SAFV-3 to pleconaril is unknown, since SAFV-3 VP1 lacks the hydrophobic pocket [22]. We therefore examined the antiviral effect of pleconaril on SAFV-3 using the SAF/UnaG virus. We pretreated SAF/UnaG with increasing amounts of pleconaril and infected HeLa cells at a multiplicity of infection (MOI) of 1, then washed the cells and incubated them in fresh medium for an additional 16 h. At 19 h post-infection, when the CPE was still not observed, we evaluated the antiviral effect of pleconaril by examining the number of UnaG-positive cells. We found that pleconaril decreased the number of UnaG-positive cells at concentrations of 40 and 50 µg/ml, demonstrating that pleconaril inhibits SAFV-3 infection in HeLa cells (Fig. 5). In addition, the antiviral effect of pleconaril was easily evaluated by UnaG fluorescence, which would—for example—enable high-throughput anti-SAFV drug screening using a fluorescence microplate reader.

As for the mechanism of the pleconaril action on SAFV-3, Mullapudi et al. reported that the space between the two β-sheets of VP1, which in some enteroviruses contains a hydrophobic cavity with a pocket factor, is filled with the side chains of amino acid residues forming the protein core in SAFV-3, and noted that it is unlikely that pocket binding inhibitors, such as pleconaril, could be used to inhibit SAFV infection [22]. In the present study, however, pretreatment with pleconaril inhibited SAFV-3 infection, suggesting that the viral attachment and/or uncoating may be prevented similarly to enteroviruses. The pleconaril may be able to bypass the steric hindrance by the CD-loop and bind to the pocket.

## Conclusions

We generated a recombinant SAFV-3 expressing GFP or UnaG as a reporter gene and demonstrated that UnaG is a superior marker to GFP because of its genetic stability during passaging. We have also shown that the SAF/UnaG virus is a useful tool for comparing SAFV susceptibility in various cell lines and evaluating the effect of antivirals on SAFV infection. Recently, UnaG has been used to generate several reporter-expressing recombinant viruses, such as rotavirus in the form of NSP3-UnaG fusion protein and adeno associated reporter virus [23, 24]. Thus, in addition to SAFV and other *Picornaviridae* family viruses, UnaG will become a promising reporter protein for the visualization of the infection of a wide variety of viruses; UnaG-expressing recombinant viruses will be a valuable tool in qualitative preliminary screening of anti-viral drugs.

## Methods

### Cell culture

HeLa cells (a human cervical cancer epithelial cell line), HCT116 cells (a human colorectal carcinoma cell line), U2OS cells (a human osteosarcoma cell line) and RAW264.7 cells (a mouse macrophage-like cell line) were maintained in Dulbecco’s modified Eagle’s medium (Sigma-Aldrich) supplemented with 10% fetal calf serum (FCS), 100 U/ml of penicillin and 100 mg/ml of streptomycin. HMV-II cells (a human malignant melanoma cell line) were maintained in RPMI 1640 medium (Wako) supplemented with 10% FCS, 100 U/ml of penicillin and 100 mg/ml of streptomycin. BHK-21 cells (a baby hamster kidney fibroblast cell line) were maintained in Eagle’s minimum essential medium (MEM, Nissui) supplemented with 5% newborn calf serum and 0.03% l-glutamine. Caco-2 cells (a human colon carcinoma cell line) were maintained in MEM supplemented with 20% FCS, 0.03% l-glutamine and 1× MEM non-essential amino acids (Gibco). CHO-K1 cells (a Chinese hamster ovary cell line) were maintained in Ham’s F12 medium (Sigma-Aldrich) supplemented with 10% FCS, 100 U/ml of penicillin and 100 mg/ml of streptomycin. All cell lines were maintained at 37 °C in a 5% CO_2_ incubator.

### Plasmid construction

The open reading frame of UnaG (GenBank accession no. AB763906) was synthesized as a DNA fragment (417 bp) by Integrated DNA Technologies (IDT) and the PCR-amplified UnaG fragment was inserted into the pBluescriptII SK(+) (Agilent) between *Xho*I and *Hind*III sites, generating pUnaG/pBSKII(+).

To construct plasmids for recombinant SAFV-3 expressing reporter proteins, the UnaG and EGFP genes with partial 2A and 2B sequences were amplified by PCR using pUnaG/pBSKII (+) and pEGFP-N1 (Clontech) as templates, respectively. The resulting PCR fragments consisted of three portions: the reporter gene without the initiating codon, a partial 2B sequence encoding PVQS at the 5’ end, and a partial 2 A sequence encoding DIETNPG at the 3´ ends. Besides, two fragments of SAFV-3 (JPN08-404) sequence, nt 3804 to 4206 and nt 4171 to 4972 (numbering according to GenBank accession no. HQ902242), were amplified by PCR using SAFV-3 infectious full-length cDNA clone pSAF404 [8] as a template. The three separate fragments were combined by overlap extension PCR. The resulting PCR products were digested with *Nco*I and *Cla*I and inserted into the pSAF404 digested with the same restriction enzymes. These recombinant plasmids were sequenced and designated as pSAF/UnaG and pSAF/GFP.

### In vitro transcription and virus generation

pSAF/UnaG and pSAF/GFP were linearized with *Not*I, and infectious RNA transcripts were synthesized using a CUGA 7 in vitro Transcription Kit (NIPPON GENE) to avoid termination of RNA transcription at the human preproparathyroid hormone (PTH) signal in the genome of SAFV-3 (JPN08-404), as described previously [8]. To generate recombinant viruses, BHK-21 cells were seeded at a density of 3.0 × 10^5^ cells per 35-mm dish. On the following day, the transcripts (10 µg) were transfected into the cells using Lipofectamine 2000 (Thermo Fisher Scientific, Inc.) according to the manufacturer’s instructions. At 20 h post-transfection, cells and supernatants were collected, and viruses were prepared by three freezing and thawing cycles to release virions, followed by centrifugation to remove cell debris. These clarified supernatant stocks from BHK-21 cells were designated as passage 0 (P0). Thereafter, virus was propagated in HeLa cells, and the lysate was prepared by three freezing and thawing cycles. Viral titers were determined by a standard plaque assay on HeLa cells.

### Fluorescence imaging of SAF/UnaG or SAF/GFP virus infected cells

The expression of the reporter gene in infected cells was captured using a JULI™ smart fluorescent cell analyzer (Digital Bio) or an EVOS M5000 Imaging System (Thermo Fisher Scientific), with nuclear staining using Hoechst 33342 when necessary. Fluorescence intensity was measured and analyzed using EVOS M5000 software.

### Genetic stability assay of recombinant viruses

SAF/UnaG virus or SAF/GFP virus was serially passaged in HeLa cells five times. The cells and supernatants were collected form infected cells, and were then frozen and thawed three times in their medium, followed by centrifugation to remove cell debris. Virus in clarified supernatant was serially passaged five times through sequential infection of HeLa cells. Viral RNAs were extracted from the clarified supernatant of the first, third and fifth passages (P1, P3, P5) of SAF/UnaG and P3 of SAF/GFP using an RNeasy Mini Kit (Qiagen). The first-strand cDNAs were synthesized using ReverTra Ace reverse transcriptase (TOYOBO) and primer for SAFV-3 (5′-CTTTCATCTACAGAATTCCAACG-3′). The obtained cDNAs were subjected to PCR as a template using KOD-plus-Neo DNA polymerase (TOYOBO) and primers for SAFV-3, which anneal to the outside of the reporter gene. The following primer pairs were used: GFP forward: 5′-TCAATCTACAGAGTTGATTTGTTC-3′ and reverse: 5′-CTCTGGTGAATTCAGTACAGTC-3′, UnaG forward: 5′-GCCTCTTATTACAAACAAAGACTCCAAC-3′ and reverse: 5′-ATTTAGTTAGCACCCCACCTTGCAACTG-3′. The amplified PCR products were analyzed on 1% agarose gels.

### Growth kinetics of recombinant viruses

Growth kinetics for SAF/UnaG and wild-type recombinant SAFV-3 (SAF404) viruses was assessed in HeLa cells. Cells were seeded at a density of 5 × 10^5^ cells/well in a 6-well plate and incubated for 24 h, followed by inoculation of each virus at a multiplicity of infection (MOI) of 5. After virus adsorption at 37 °C for 60 min, they were washed twice with Dulbecco’s phosphate buffered saline (PBS), and incubated at 37 °C in DMEM with 1% FCS. The cells and supernatants of each culture were collected at 0, 3, 6, 10, 24, and 48 h post-infection. The cell-associated viruses were prepared by three freezing and thawing cycles from the cells. Cell-free and cell-associated viruses were titrated by a standard plaque assay on HeLa cells.

### Immunofluorescence staining

For immunofluorescence staining of SAF404-infected cells, cells were grown on glass coverslips and infected with SAF404 at 37 ˚C. The cells were fixed with 4% paraformaldehyde for 20 min at 4 ˚C, permeabilized with 0.2% Triton X-100 for 15 min at room temperature, and blocked with 5% skim milk in PBS-T for 60 min at room temperature. Subsequently, cells were incubated overnight at 4 ˚C with anti-SAFV-3 antiserum [20], washed with PBS-T, and then incubated with Alexa Fluor 488-conjugated anti-rabbit IgG (Thermo Fisher Scientific) for 60 min at room temperature. Coverslips were mounted using ProLong Diamond Antifade Mountant with DAPI (Thermo Fisher Scientific). Images were captured using an Olympus IX73 fluorescence microscope (Olympus), and the number of infected cells was quantified using ImageJ software.

### Antiviral assay of pleconaril against SAF/UnaG

Pleconaril was obtained from FUJIFILM Wako Pure Chemical. Pleconaril was dissolved in dimethyl sulfoxide (DMSO). HeLa cells were seeded at a density of 4.4 × 10^4^ cells/well in a 48-well plate and incubated for 24 h. Various concentrations of pleconaril (0, 0.1, 1, 10, 20, 30, 40, and 50 µg/ml) were mixed with SAF/UnaG virus (2.75 × 10^5^ PFU/550 µl). Pleconaril/virus mixtures were prepared so that the final DMSO concentration was 0.5% in each well and then incubated for 60 min at room temperature prior to infection. HeLa cells were infected with pretreated SAF/UnaG virus at an MOI of 1 (1.25 × 10^5^ PFU/well). After virus adsorption at 37 °C for 3 h, they were washed twice with PBS, and incubated at 37 °C in DMEM with 10% FCS. At 16 h, UnaG-expressing cells were photographed using a JULI™ smart fluorescent cell analyzer. The antiviral activity of pleconaril was evaluated by number of cells with UnaG fluorescence.

### Statistical analysis

Statistical analysis was performed with GraphPad Prism software version 7 (GraphPad Software).

### Electronic supplementary material

Below is the link to the electronic supplementary material.

Additional File 1: Unedited gel images of Figures 2A and B. The dotted lines show the cropped areas used in Figure 2

## Data Availability

Not applicable.
