# Supplementary figures and images for: Generation of a recombinant Saffold Virus expressing UnaG as a marker for the visualization of viral infection
Source: Virol J. 2023 Aug 7;20:175. doi: 10.1186/s12985-023-02142-8 (PMC10408109; doi:10.1186/s12985-023-02142-8)

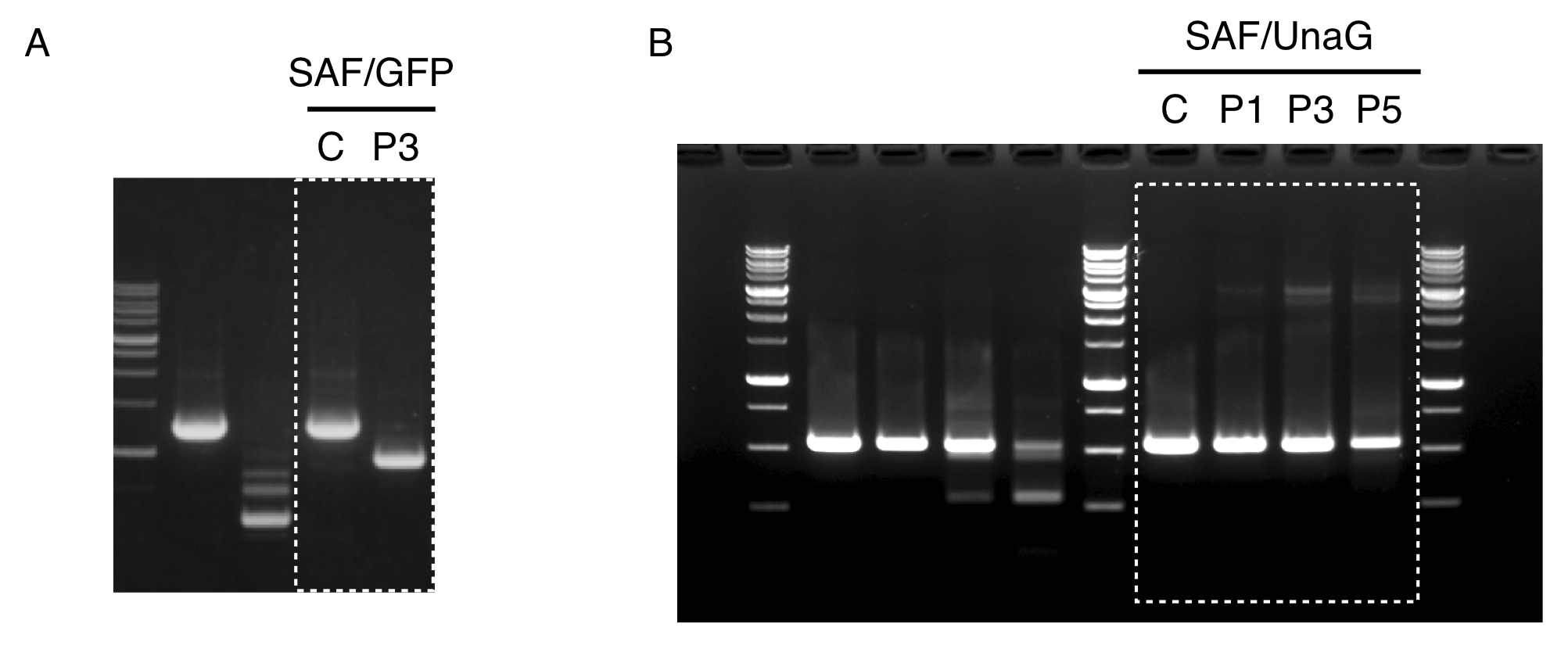

Supplement: Supplementary file 1 — Additional File 1: Unedited gel images of Figures 2A and B. The dotted lines show the cropped areas used in Figure 2 [file 12985_2023_2142_MOESM1_ESM.tiff]
